# Stress and stem cells: adult Muse cells tolerate extensive genotoxic stimuli better than mesenchymal stromal cells

**DOI:** 10.18632/oncotarget.25039

**Published:** 2018-04-10

**Authors:** Nicola Alessio, Tiziana Squillaro, Servet Özcan, Giovanni Di Bernardo, Massimo Venditti, Mariarosa Melone, Gianfranco Peluso, Umberto Galderisi

**Affiliations:** ^1^ Genome and Stem Cell Center (GENKOK), Erciyes University, Kayseri, Turkey; ^2^ Department of Experimental Medicine, Campania University “Luigi Vanvitelli,” Naples, Italy; ^3^ Sbarro Institute for Cancer Research and Molecular Medicine, Center for Biotechnology, Temple University, Philadelphia, PA, USA; ^4^ Institute of Bioscience and Bioresources, CNR, Naples, Italy; ^5^ 2nd Division of Neurology, Center for Rare Diseases & InterUniversity Center for Research in Neurosciences, Department of Medical, Surgical, Neurological, Metabolic Sciences, and Aging, University of Campania "Luigi Vanvitelli", Napoli, Italy

**Keywords:** mesenchymal stem cells, senescence, apoptosis

## Abstract

Mesenchymal stromal cells (MSCs) are not a homogenous population but comprehend several cell types, such as stem cells, progenitor cells, fibroblasts, and other types of cells. Among these is a population of pluripotent stem cells, which represent around 1–3% of MSCs. These cells, named multilineage-differentiating stress enduring (Muse) cells, are stress-tolerant cells.

Stem cells may undergo several rounds of intrinsic and extrinsic stresses due to their long life and must have a robust and effective DNA damage checkpoint and DNA repair mechanism, which, following a genotoxic episode, promote the complete recovery of cells rather than triggering senescence and/or apoptosis.

We evaluated how Muse cells can cope with DNA damaging stress in comparison with MSCs. We found that Muse cells were resistant to chemical and physical genotoxic stresses better than non-Muse cells. Indeed, the level of senescence and apoptosis was lower in Muse cells. Our results proved that the DNA damage repair system (DDR) was properly activated following injury in Muse cells. While in non-Muse cells some anomalies may have occurred because, in some cases, the activation of the DDR persisted by 48 hr post damage, in others no activation took place.

In Muse cells, the non-homologous end joining (NHEJ) enzymatic activity increases compared to other cells, while single-strand repair activity (NER, BER) does not. In conclusion, the high ability of Muse cells to cope with genotoxic stress is related to their quick and efficient sensing of DNA damage and activation of DNA repair systems.

## INTRODUCTION

Mesenchymal stromal cells (MSCs) are present in the stroma of several organs and tissues, such as bone marrow, adipose tissue, dental pulp, and umbilical cord. MSCs are not a homogenous population but comprehend several cell types, such as stem cells, progenitor cells, fibroblasts, and other types of cells [1].

Stem cells present in an MSC population can differentiate into mesodermal lineage cells (adipocytes, chondrocytes, osteocytes, and muscle cells) but also in cells belonging to endodermal and ectodermal lineages, at least *in vitro* [2]. For this reason, several researchers proposed that MSCs may contain a subpopulation of pluripotent stem cells. Indeed, in the past, several authors have identified putative pluripotent stem cells in MSCs, such as multipotent adult progenitor cells (MAPCs) or very small embryonic stem cells (VSELs). Many scientists questioned the existence of these cells. In recent years, the Dezawa’s research group identified a population of pluripotent stem cells, which represent around 1–3% of MSCs. These cells were named multilineage-differentiating stress enduring (Muse) cells since they were found to be stress-tolerant cells. Muse cells express the pluripotent surface marker SSEA-3 and other pluripotency genes (NANOG, OCT-3/4, SOX2). They can differentiate into triploblastic cells from a single cell and are self-renewable [2, 3].

In MSC cultures, other cell types do not possess the properties of Muse cells [4]. Indeed, Muse cells, isolated from a heterogeneous stromal cell culture, can differentiate into functional melanocytes, while non-Muse cells fail to do so [5]. In an animal model of stroke, Muse cells can replenish lost neurons and contribute to pyramidal tract reconstruction [6]. Muse cells can also differentiate into liver cells when intravenously injected into animals that were subjected to hepatectomy [7, 8]. All these studies indicate that Muse cells are pluripotent, but non-Muse cells in MSC cultures are not.

During the lifetime of an organism, cells, which form tissues and organs, experience several types of intrinsic and extrinsic stresses. Metabolic functions with reactive oxygen production and DNA replication are among the main intrinsic stressors, while chemical and physical genotoxic events are the environmental factors that may negatively affect a cell’s activities. Following a DNA damage occurrence, cells trigger events aimed at eliminating and/or reducing the possibility that injured cells will experience a neoplastic transformation. Specific stress responses imply a correct DNA repair to completely recover performances of damaged cells [9]. Alternatively, cells harboring unrepairable damages may enter apoptosis or senescence [10, 11].

Stem cells may undergo several rounds of intrinsic and extrinsic stresses due to their long life. On the other hand, they must preserve their full functionality to promote tissue and organ homeostasis. For this reason, stem cells must have a robust and effective DNA damage checkpoint and DNA repair mechanism, which, following a genotoxic episode, promote the complete recovery of cells rather than triggering senescence and/or apoptosis [9]. We could assert that the more a stem cell is stress tolerant with an accurate DNA repair system, the better it could play a key role in body homeostasis.

On this premise, we decided to evaluate how Muse cells cope with DNA damaging stress compared with MSCs. We treated cells with chemical and physical stressors and evaluated activation of DNA damage checkpoint and repair capacity. We also determined the level of senescence and apoptosis.

## RESULTS

### Muse cells were resistant to genotoxic stresses

Our comparison study was carried out on a global MSCs and their SSEA-3-positive (Muse cells) and negative (non-Muse cells) subpopulations. On these cells, we evaluated the level of apoptosis and senescence following chemical and physical genotoxic stress, that is, peroxide hydrogen (H_2_O_2_) treatment and UV irradiation, respectively. Apoptosis may occur soon after DNA damage while the triggering of senescence requires longer time. For this reason, we evaluated apoptosis 1 and 48 hr post-treatment, whereas senescence was determined only at 48 hr.

**Figure 1:**
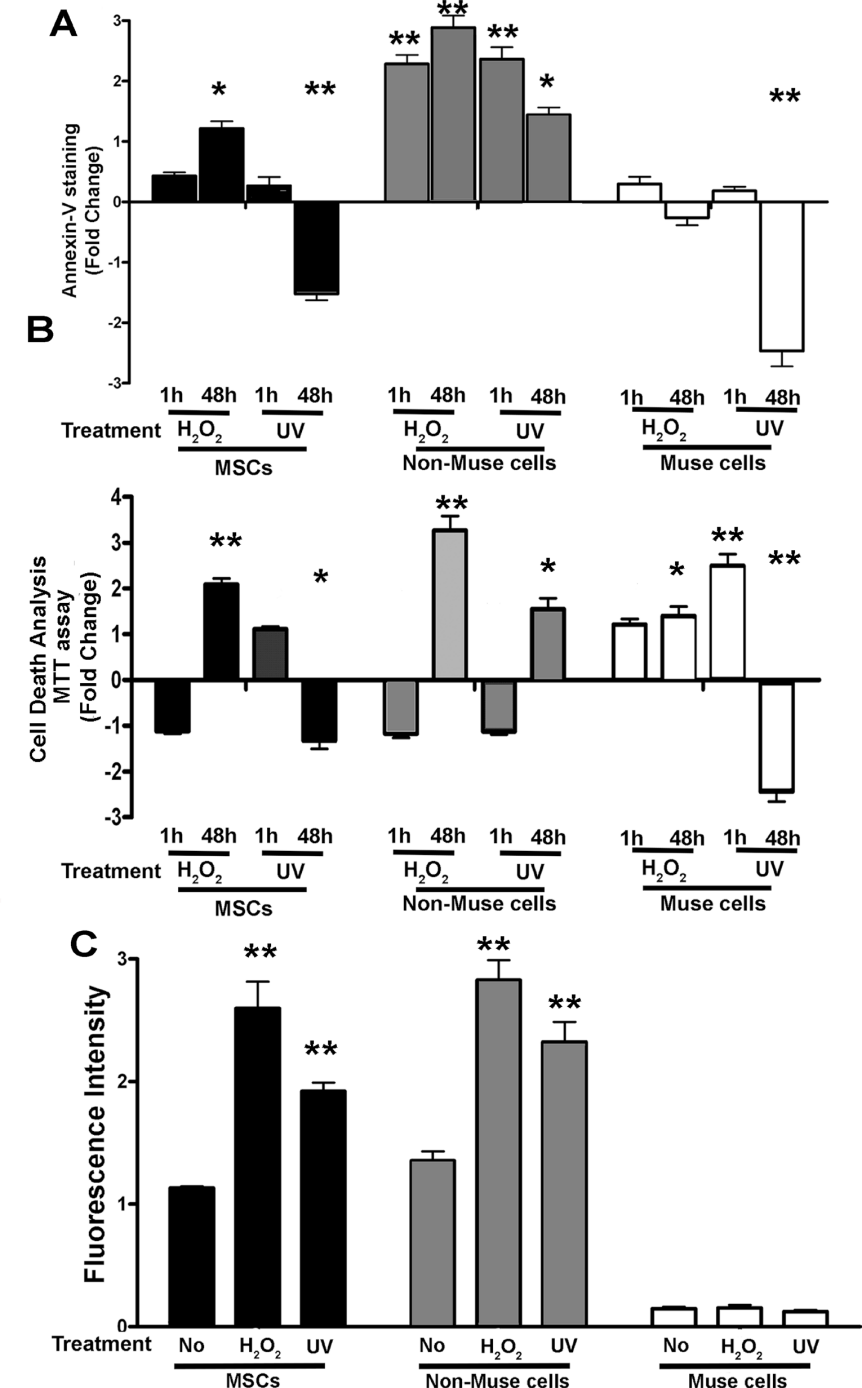
Cell death and senescence (**A**, **B**) Apoptosis and global cell death analysis, performed with Annexin V and formazan assays, respectively. Data are expressed as fold changes with standard error (± SD, *n* = 3, ^*^p < 0.05, ^**^p < 0.05). For each cell type, in every experimental condition, the apoptosis or global cell death level in samples not treated with genotoxic agents (H_2_O_2_ or UV) is set as the baseline (zero value). Increase or decrease of apoptosis or cell death in treated samples is shown as columns above or below the control baselines, respectively. (**C**) Acid beta-galactosidase senescence assay. The graph shows senescent level in every experimental condition (± SD, *n* = 3, ^*^p < 0.05). Data expressed as arbitrary units (fluorescence intensity).

In MSCs, we detected an increase in apoptosis 48 hr after treatment with peroxide hydrogen while we observed no change 1 hr following UV irradiation and a decline at 48 hr (Figure 1A). In non-Muse cells, we detected an increase in apoptosis 1 hr following incubation with either peroxide hydrogen or UV treatment. This phenomenon persisted at 48 hr (Figure 1A). In Muse cells, neither treatment produced increments in apoptosis; moreover, UV irradiation induced a diminution of apoptosis 48 hr post-treatment (Figure 1A).

The viability assay allowed us to determine the increase in cell death (including necrosis) following each cytotoxic treatment. In the three cell populations (MSCs, non-Muse, and Muse), we detected an increase in cell death after either peroxide hydrogen or UV treatment (Figure 1B). This phenomenon occurred either soon after genotoxic injury (1 hr) or later on (48 hr). In particular, non-Muse cells showed the highest percentage of cell death (Figure 1B).

The percentage of senescent cells increased in MSCs and non-Muse cells after incubation with peroxide hydrogen while Muse cells were unaffected. The UV irradiation produced a minimal augment of senescence only in non-Muse cells (Figure 1C).

### Activation of DNA damage detection and repair following stress

DNA injury triggers the activation of the DDR system. This activation takes place after damage and may persist for some hours. The first step is the cell cycle arrest to ensure that the damage is repaired before the cell cycle restarts. Cells may arrest at the G_1_/S or G_2_/M transition phases.

**Figure 2:**
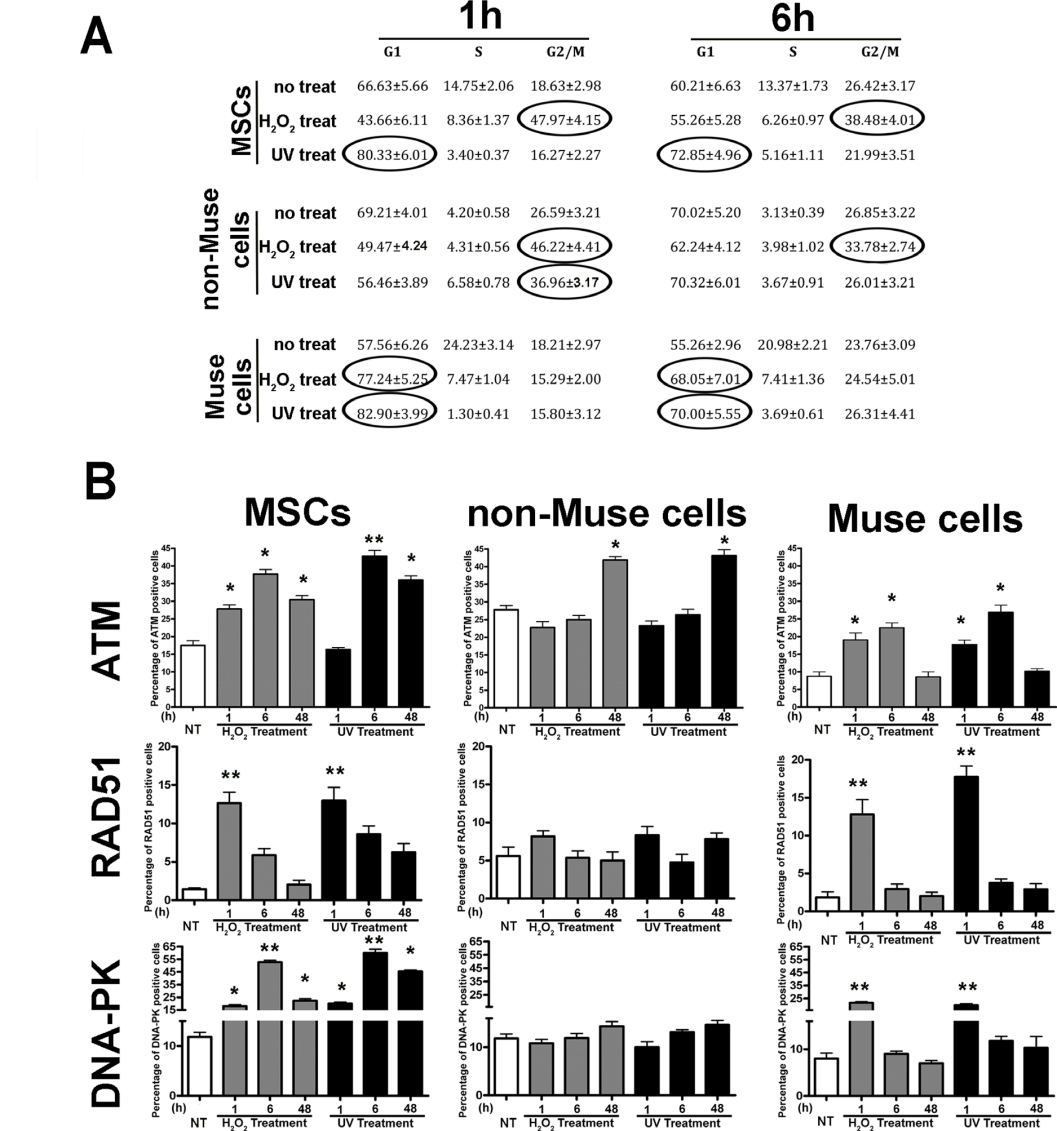
Cell cycle and DNA repair signaling MSCs, non-Muse cells, and-Muse cells were treated with H202 or UV. (**A**) - The table shows the cell cycle analysis 1, 6, and 48 hr following treatments. Data are expressed in percentage (± SD, *n* = 3). In treated samples, the values in the ovals are statistically different (*p* < 0.05) from corresponding controls. (**B**) - The graphs show results from the immunostaining analysis of proteins involved in DNA repair signaling 1, 6, and 48 hr following treatments. The mean percentages of ATM, RAD51, and DNA-PK positive cells are reported (± SD, *n* = 3, ^*^p < 0.05, ^**^p < 0.05).

We then performed analyses on the DDR at 1, 6, and 48 hr post-genotoxic treatment. At the 1 and 6 hr post-genotoxic treatment, MSCs arrested either at G_2_/M (peroxide hydrogen treated) or G_1_/S (UV irradiated) (Figure 2A). Then, at 48 hr, cell cycle resumed, and its pattern was not statistically different from that of the control cultures. Non-Muse cells arrested at the G_2_/M boundary irrespective of genotoxic agent (Figure 2A). At 48 hr, post-damage cells resumed a normal cycling pattern. Muse cells were locked in at G_1_ following DNA damage events both 1 and 6 hr post-injury (Figure 2A). Also, in this case, cells regained normal cycling activity at 48 hr following damages. Collectively, this data demonstrated that DNA damage checkpoints effectively worked in the different cell populations.

Following DNA injury, cell cycle arrest is associated with induction of DNA repair machinery. Activation of ataxia telangiectasia mutated kinase (ATM), which regulates the DNA repair process, occurs soon after DNA damage. This protein binds DNA-damage foci and contributes to recruitment of DNA repair enzymes. After that, ATM is inactivated and dissociates from the foci [12, 13].

Both H_2_O_2_ and UV irradiation produced a significant increase in the number of ATM(+) cells 6 hr post-treatment in MSCs. Then, 48 hr following injury, the ATM(+) cells decreased but were still significantly higher than those observed in control cultures (Figure 2B). In non-Muse samples, the percentage of cells having activated ATM increased only 48 hr post genotoxic treatment (Figure 2B). In Muse cells, we detected an increase in ATM(+) 1 and 6 hr post damage. This augment, however, was lower than those evidenced in MSCs and non-Muse cells. Furthermore, 48 hr after stress, the number of ATM(+) cells declined to the control level (Figure 2B).

We further evaluated the response of the DDR system by analyzing the expression of key downstream effectors of ATM, that is, RAD51 and DNA-PK. These two proteins are involved in the activation of homologous (HR) and non-homologous-end-joining (NHEJ) DNA repair systems, respectively [13–15].

One hour after peroxide hydrogen treatment, RAD-51(+) cells increased significantly in the MSC culture. Then, this percentage declined at the basal value. Irradiation of MSCs with a UV-induced augment of RAD-51(+) cells. This increase did not decline to the basal level even 48 hr post-treatment (Figure 2C). In non-Muse cells, we did not observe any changes in the percentage of RAD-51(+) cells, which implied that the cell machinery did not react to genotoxic insults (Figure 2C). In Muse cells, the incubation with H_2_O_2_ or UV irradiation induced an increase of RAD-51(+) cells soon after treatment and then a later decline in basal levels (Figure 2C).

In MSCs, we detected a significant increase of DNA-PK positive cells following H_2_O_2_ treatment and UV irradiation. The upregulation of DNA-PK expression peaked by 6 hr post DNA injury and then declined by 48 hr but did not reach the basal value observed in the control samples (Figure 2D). Of interest, in non-Muse cells, we did not evidence any changes in the percentage of DNA-PK(+) cells following peroxide or UV treatment, which implied that the system did not respond properly to DNA damage (Figure 2D). This is in agreement with impairment of ATM activation, which should occur soon after genotoxic insult and not 48 hr later. Muse cells evidenced an increase in DNA-PK(+) cells by 1 hr post stress, and then this level declined to the basal value (Figure 2D).

### Persistence of unrepaired DNA in MSCs and non-Muse cells following genomic injury

We analyzed the expression level of γ-H2AX in order to evaluate the repair capacity of cells following genotoxic stress. The histone H2AX is phosphorylated following activation of ATM, and this phosphorylated isoform (γ-H2AX) is implicated in the enrollment and/or retention of DNA repair proteins. The foci of γ-H2AX in nuclei are signs of damaged DNA that are subjected to repair. Soon after DNA injury, the presence of these foci is a sign of active repair. On the other hand, foci permanency several hours or days after stress stimuli indicates the presence of unrepaired or misrepaired DNA [16, 17].

We performed a flow cytometry analysis of γ-H2AX protein, combined with DNA staining, to detect foci of damaged DNA in the different cycle phases [18].

**Figure 3:**
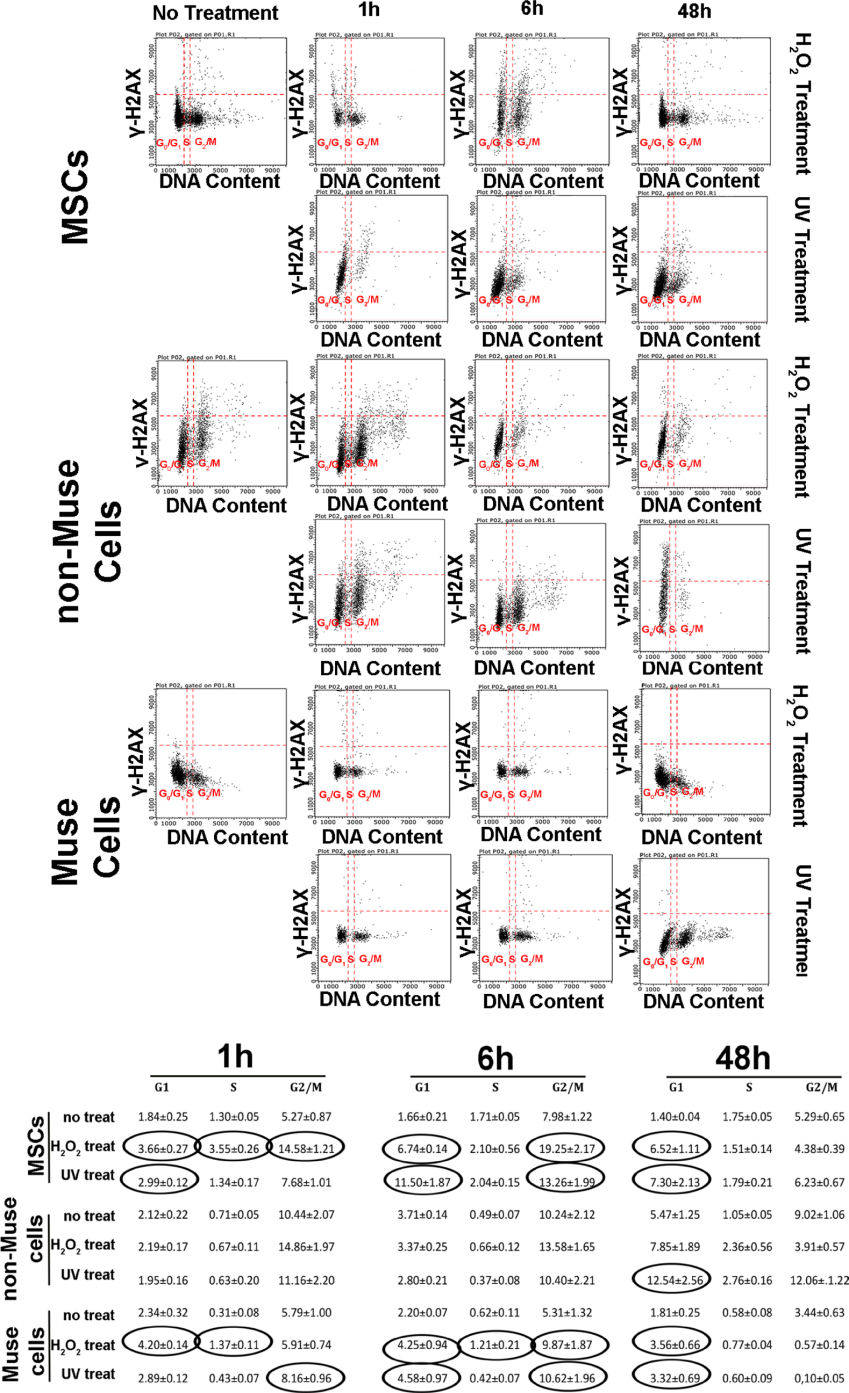
DNA damage and cell cycle MSCs, non-Muse cells, and-Muse cells were treated with H202 or UV. At 1, 6, and 48 hr following treatments, a flow cytometry analysis of γ-H2AX protein, combined with propidium iodide DNA staining, was performed to detect foci of damaged DNA in the different phases of the cycle. For every experimental condition, the percentages of γ-H2AX-positive cells are indicated in the table. Data are expressed with standard deviation (*n* = 3). In treated samples, the values in the ovals are statistically different (*p* < 0.05) from corresponding controls.

We observed a very significant increment of γ-H2AX staining in MSCs treated with both stressors (Figure 3). At 1 and 6 hr post-DNA injury, this increment was detected mainly in cells traversing the G_2_/M phase for peroxide hydrogen-treated cultures, while it was present in G_1_ cells following UV irradiation (Figure 3). At 48 hr, the g-H2AX foci persisted in the G_1_ phase, and no decline in the basal level occurred. Non-Muse cells showed an anomalous behavior following DNA damage. We did not observe any increase in g-H2AX staining at both 1 and 6 hr post-injury (Figure 3). The increment was detected in G_1_ cells at 48 hr (Figure 3).

Muse cells presented an upregulation of γ-H2AX staining 1 and 6 hr post-treatment. This occurred either in cells traversing the G_1_, S, or G_2_/M phase for peroxide hydrogen-treated cells, while it was present only in G_1_ or G_2_/M cells following UV irradiation (Figure 3). We still detected γ-H2AX foci in G_1_ cells at 48 hr (Figure 3). Of note, this percentage was two or more times lower than those observed in MSCs and non-Muse cells at 48 hr.

### Difference in the efficiency of repair systems among MSCs, non-Muse, and Muse cells

The cell’s capacity for repairing DNA is linked to enzymatic activity of DNA repair enzymes. We then evaluated the ability of Muse cells to repair DNA damage compared with non-Muse cells and MSCs. We analyzed in detail the proficiency in repairing DNA double- strand breaks by either NHEJ by a plasmid-based assay. We also evaluated the cell’s skill in repairing a single-strand DNA break by BER and NER.

**Figure 4:**
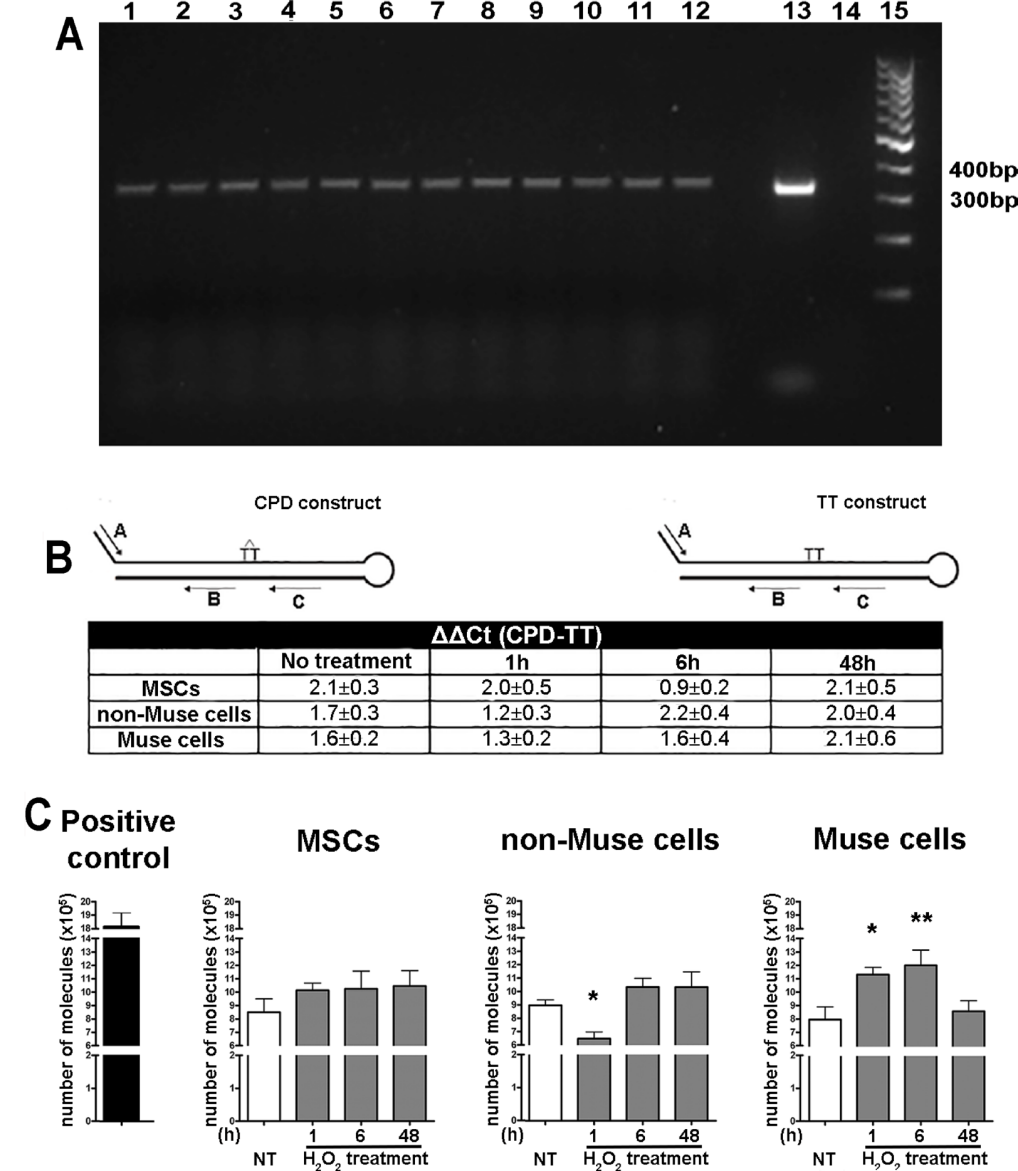
Enzymatic assays for detection of DNA repair proficiency MSCs, non-Muse cells, and-Muse cells were treated with UV (**A**, **B**) or H_2_0_2_ (**C**). 1, 6, and 48 hr following treatments, several enzymatic assays were carried out for evaluation of DNA repair abilities. Panel A: A 347 bp fragment of human GAPDH gene was amplified by PCR and analyzed by agarose gel electrophoresis following incubation with cell lysates. DNA amplicons contained uridine deoxynucleotides and could be degraded by uracil DNA glycosidase present in cell extracts. This enzymatic activity was indirectly determined by calculating the ratio between pixel intensities of intact DNA bands coming from cell lysate- treated and untreated amplicons. In the panel is reported an exemplificative agarose gel. GAPDH amplicons were incubated with untreated MSCs (lane 1) or with MSC cell extracts obtained 1 hr (lane 4), 6 hr (lane 7), 48 hr (lane 10) following UV irradiation. Same experimental scheme was applied for non-Muse cells (lanes 2, 5, 8, 11) and Muse cells (lanes 3, 6, 9, 12). Lane 13: GAPDH amplicon not incubated with cell extracts (BER negative control); lane 14: GAPDH amplicon treated with uracil DNA glycosidase (BER positive control); lane 15: DNA molecular weight marker. (B) Two different DNA constructs with a hairpin duplex structure (CPD and TT construct, respectively) were used as a template for qPCR amplification (see inset). CPD and TT constructs differ only in a region that has either a normal TT couple (TT construct) or a cyclobutane pyrimidine dimer (CPD construct). Two primers, named A and C, encompassed a region containing cyclobutane pyrimidine dimers in the CPD construct and a normal TT couple in the TT construct. The presence of these dimers in the CPD construct impaired activity of DNA polymerase during PCR unless these lesions were repaired by the NER system. Two other primers, named A and B, encompassed a DNA region that did not contain dimers in both constructs and served as controls of PCR efficiency. The constructs were incubated with cell lysates from Muse cells, non-Muse cells, and MSCs. In these conditions, the NER activity of cell extracts might repair a cyclobutane dimer and increase PCR yield in samples containing the CPD construct as templates. The incubation of the TT construct with cell lysate did not further increase PCR products. Data analysis were performed with the following equation: DCt = Ct _(A/C primers)_ – Ct _(A/B primers)._ The DCt was determined for TT construct, used as reference, and for CPD construct. We calculated the NER activity in each experimental condition with DDCt value (CPD – TT construct). The table reports the NER activity before (No treatment) and after (1, 6, 48 hr) UV treatment (± SD, n = 3). (C) The p100048 plasmid was digested with an Age I enzyme and used as the template for a qPCR reaction that amplifies a 135 bp fragment encompassing the region containing the Age I restriction site. Digested plasmids were incubated with cell lysates from Muse cells, non-Muse cells, and MSCs. In these conditions, the NHEJ activity of cell extracts might end-join plasmids and allow PCR amplification. Different amounts of undigested plasmids were amplified to create a standard curve with Ct (threshold cycle). This allowed us to obtain a relative measure of the amount of amplicon in the different experimental conditions. The higher the quantity of obtained amplicon from digested plasmid templates, the better the NHEJ activity. The graphs named MSCs, non-Muse cells and Muse cells report the number of amplified molecules before (NT) and after (1, 6, 48 hr) H_2_O_2_ treatment (± SD, *n* = 3, ^*^p < 0.05, ^**^p < 0.01). Positive control shows the number of amplified molecules obtained from undigested plasmid.

In BER pathways, DNA glycosylases are the key enzymes. They remove damaged or aberrant purine/pyrimidine bases from DNA. We incubated cellular extracts with DNA fragments containing uracil-deoxynucleotides to induce excision of these aberrant bases by DNA glycosylase. This *in vitro* enzymatic activity induces DNA fragmentation that can be visualized as a smear following DNA electrophoresis. We obtained cellular extracts either from cells cultivated in normal conditions (naïve conditions) or following DNA damage that should further activate a cell’s DNA repair system (primed conditions). We selected UV irradiation as genotoxic agent, since it may cause single nucleotide mutations that can be substrate for BER. In all the tested experimental conditions, we did not detect any significant difference in BER activity among MSCs, non-Muse cells, and Muse cells (Figure 4). It should be underlined that primed cells showed increased BER activity with respect to naïve cells in all three cell types.

The assay to detect NER enzymatic activity relies on the ability of repair enzymes to remove CPD dimers from a DNA template. The higher the DNA repair enzymatic activity, the more efficiently the Taq DNA polymerase works. Also in these experiments we used UV treated cells, since this treatment produces cyclobutane pyrimidine dimers, (6–4) photoproducts, which are a typical NER substrate. The assay showed that Ct values of qPCR for each experimental condition did not differ in a statistically significant manner. This indicates that proficiency in repairing DNA with the NER system is equivalent among Muse cells, non-Muse cells, and MSCs (Figure 4).

The assay to detect NHEJ uses an Age I digested plasmid as the template for a qPCR reaction. PCR amplification occurs on plasmids that had been end-joined by NHEJ activity present in cell extracts. We used H_2_0_2_ primed cells, since this damaging agent may induce double strand breaks that can be repaired by NHEJ. The plasmid assay evidenced that Muse cells had a higher NHEJ activity compared with non-Muse cells and MSCs (Figure 4).

### Genotoxic events induced changes in the expression levels of key genes involved in DNA repair

A cell experiencing DNA damage may trigger a quick response by activating the repair proteins already present in the nucleus. Given the presence of stressful environmental conditions, a cell may also activate a late response to be prepared for future harmful stimuli. To this end, a cell may induce changes in mRNA levels of genes involved in DNA repair systems.

We then evaluated if, following DNA damage, Muse cells modified the mRNA expression of representative genes involved in NER, BER, mismatch repair (MMR), NHEJ and homologous recombination (HR) [19–21].

**Figure 5:**
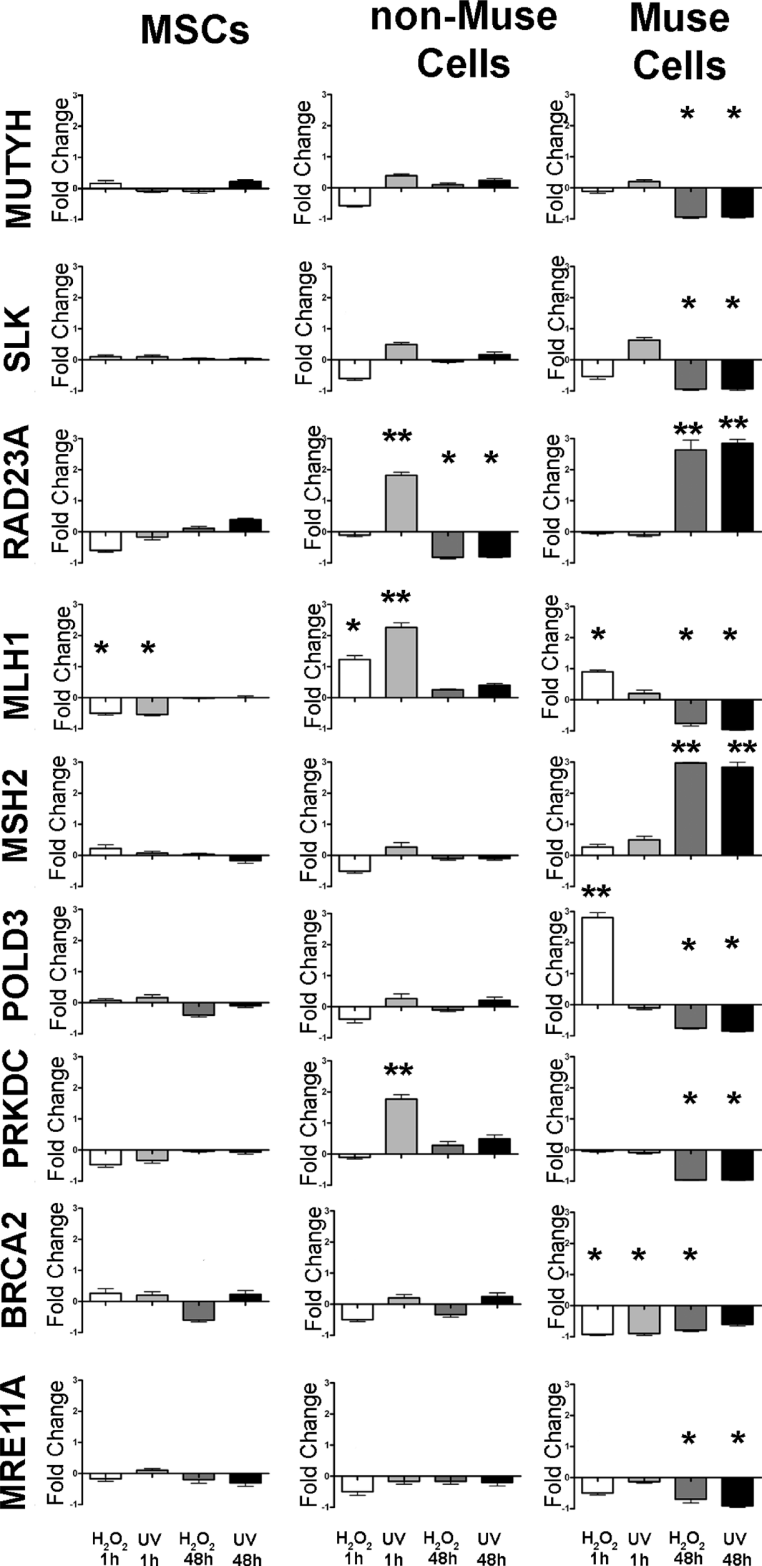
mRNA expression levels of BER, NER, MMR, NHEJ, and HR genes The histograms show the quantitative RT-PCR analysis of a group of genes involved in BER, NER, MMR, NHEJ, and HR. The mRNA levels were normalized to GAPDH mRNA expression, which was selected as an internal control. Histograms show expression levels in the different conditions. Data are expressed as fold changes with standard error (± SD, *n* = 3, ^*^p < 0.05). For each gene in every experimental condition, the expression level in not-differentiated samples is set as the baseline (one value). Up- or down-regulation of genes in differentiated samples is shown as columns above or below the control baselines, respectively.

Soon after DNA damage, non-Muse cells showed more changes in the expression levels of DNA repair genes compared with other cell types, while at 48 hr post genotoxic stress, Muse cells evidenced significant changes in the expression of the analyzed gene (Figure 5).

## DISCUSSION

During their long life, stem cells experience several intrinsic and extrinsic stresses. For this reason, they have very active anti-stress and anti-transformation protective mechanisms that help to preserve their functionality to promote tissue and organ homeostasis. We could affirm that stress reduction mechanisms are a critical and typical feature of stem cells. The study of these phenomena is fundamental for understanding the physiological functions of stem cells as well as for advances in safe and effective stem cell-based medical treatments. In this scenario, we aimed to evaluate the ability of stem cells to survive following stressful genotoxic stimuli. Knowing stress resistance of stem cells is of paramount importance because in some diseases, such as brain stroke, myocardial infarction, or kidney failure, a massive apoptosis and degeneration of tissue cells occur. This produces a very stressful environment, and stem cell therapies may fail because stem cells may be damaged before they exert their regenerative effects.

### Muse cells showed an efficient DNA damage sensing and repair capacity

MSCs are currently applied in several therapies even if their features remain to be better characterized. Recently, Muse cells are a stem cell subpopulation in MSCs; hence, improved MSC-based treatments might be realized by the appropriate use of Muse cells.

These are pluripotent stem cells and can exert repair effects on various organs and tissues. On these premises, it is of interest to evaluate if Muse cells have a robust and effective anti-stress mechanism compared to the whole MSC population.

We found that Muse cells were more resistant to chemical and physical genotoxic stresses than non-Muse cells and the global MSC population. Indeed, the level of senescence and apoptosis was lower in Muse cells. Our results proved that the DDR is properly active following injury in Muse cells. However, in non-Muse cells and MSCs, some anomalies may have occurred because in some cases the activation of the DDR by 48 hr post-damage persisted while in others, no activation took place. These findings may indicate that only in Muse cells were DNA damages accurately repaired as confirmed by g-H2AX staining.

The cell cycle analysis associated with detection of activated H2AX (g-H2AX) evidenced that 1 hr following stress, Muse cells and MSCs with damaged DNA can be found in all the cycle phases, suggesting that repair mechanisms may work throughout the cell cycle. Presence of activated H2AX only in G_1_ cells, 48 hr post-DNA injury, is in agreement with the hypothesis that cells bearing unrepaired/misrepaired DNA arrest in G_1_ phase. Because non-Muse cells evidenced activated H2AX staining in G_1_ cells only 48 hr following stress, this further suggests that these cells were not proficient in DNA repair. Of note, Muse cells also showed some G_1_ arrested cells with unrepaired DNA 48 hr after DNA-damaging treatment. Nevertheless, the percentage of cells having activated H2AX was lower than those found in MSCs and non-Muse cells.

The effective capacity of Muse cells to repair DNA is additionally evidenced by looking at activation of RAD51 and DNA-PK, which are involved in the HR and NHEJ repair systems, respectively. Both proteins are activated soon after DNA damage and decline to the basal level 6 hr following stress. This, along with g-H2AX staining, demonstrates the existence of a quick and efficient DNA repair process. The MSCs showed activation of HR and NHEJ repair, but this process was prolonged as if the DNA was not completely repaired and the system could not shut down. The lack of activation of these repair mechanisms in non-Muse cells lends credit to the presence of a failure in their DNA repair ability.

The DNA repair process relies upon a DNA damage-sensing mechanism that recruits and activates enzymes directly involved in DNA repair (DNA helicases, nucleases, polymerases, and ligases). We then evaluated the proficiency of some of these enzymes involved in BER, NER, and NHEJ to obtain a global vision of the DNA repair capacity.

The BER and NER assay evidenced that these repair pathways had the same efficiency in Muse cells, non-Muse cells, and MSCs, whereas NHEJ activity was higher in Muse cells compared with the other cell types. These data partially overlap those reported for other pluripotent stem cells, such as embryonic stem cells (ESCs) and induced pluripotent stem cells (iPSCs). Indeed, some authors reported an increase in BER and/or NHEJ and HR activity. Conflicting reports, we showed, may be related not only to the intrinsically specific properties of pluripotent Muse cells but also to the differences in assays used to detect activation of DNA repair pathways [22–25]. Here, we used both immunocytochemistry assays to detect activation of the DNA repair mechanism and *in vitro* assays to evaluate the enzymatic ability to repair DNA damages by the BER, NER, or NHEJ repair systems. This strategy allowed us to evidence that the higher repair capacity of Muse cells compared to non-Muse cells and MSCs is related to their quick and efficient sensing of DNA damage and activation of DNA repair systems. The enzymatic activity of repairing proteins is increased only in the NHEJ system and not in single-strand repair (NER, BER). We may explain the high activity of NHEJ by considering that this is the only mechanism that can repair double-strand breaks in every phase of the cell cycle. Muse cells may have a powerful NHEJ system to survive strong genotoxic stress. On the other hand, NHEJ is an error-prone repair system that may promote the onset of mutation within the genome. This, in turn, may trigger a neoplastic transformation. This phenomenon further supports the hypothesis of tumor onset driven by mutations occurring in normal stem cells.

### DNA damages appear to trigger an adaptive response mechanism

Cells may respond to extrinsic and intrinsic stimuli by modifying gene expression at various levels: transcriptional, post-transcriptional, translational, and post-translational. Transient modifications in gene expression are mainly related to changes in the rate of protein translation and/or degradation as well as in post-translation modifications. Permanent or durable changes in gene expression are associated with changes at the transcriptional and post-transcriptional tiers. These occur when environmental conditions require a constant and long-lasting modification of the gene expression landscape. The mRNAs analysis of genes involved in the DNA repair process allowed us to evaluate if cells prepare themselves to cope with a new wave of DNA damage.

From 1 to 48 hr following DNA injury, Muse cells showed several expression changes (either up- or down-regulation) in genes involved in DNA repair. The observed modifications in the levels of DNA repair-related mRNAs may be part of the so-called adaptive response mechanism. According to scientists who hypothesized the existence of this mechanism, the induction of a repair process after a small priming dose of a DNA stressor can protect cells against a larger second dose given several hours or days later [19]. We should underline that the activation of an adaptive response does not imply that all “DNA repair genes” must be upregulated but rather that a change in the expression (increase or decrease) should occur, which happens in every complex and well-harmonized system, implying positive and negative feedbacks. As pure speculation, the onset of an adaptive response 48 hr post-injury may explain the reduced level of apoptosis in Muse cells (below control value). Several changes in the expression of genes involved in DNA repair also occurred in non-Muse cells, especially 1 hr post-injury. This may indicate that, given the inability to activate the DDR, non-Muse cells try to cope with DNA damage by inducing a quick synthesis of new proteins. This is a mere hypothesis that needs further investigation. MSCs appeared little inclined to activate an adaptive response following DNA damage events.

In conclusion, our data evidenced that Muse cells possess an efficient and rapid mechanism to cope with environmental stresses that may induce DNA damage. This is in line with the hypothesis that stem cells must be stress tolerant, with an accurate DNA repair system, since they persist for a long time in our bodies and, hence, will experience several rounds of intrinsic and extrinsic genotoxic events. The rapidity of Muse cells in detecting damaged DNA and activating repair mechanisms may explain the high efficiency of this process. Quoting a saying of ancient Rome, “Bis dat qui cito dat,” we may say: “Help rendered promptly is worth twice as much.” It remains to be seen why non-Muse cells were not able to properly activate the DNA damage pathway. This population is composed of all cell phenotypes present in MSCs except the Muse cells, which may represent a maximum 3% of the whole population. In other words, while disrupting the equilibrium among the several cell types present in MSCs positively affected the DNA repair capacity of isolated Muse cells, it greatly impairs that of non-Muse cells. Does this imply a cross-talk between Muse cells and other cell types to sense a damaging event and manage it? This is an issue that scientists should investigate to dissect paracrine signaling that may occur between stem cells and surrounding cell populations. Our study was carried out on bone marrow-derived Muse cells; hence genotoxic stress tolerance for Muse cells obtained by other stromal sources remains to be determined. Current knowledge, however, evidences that Muse cells have the some biological features irrespective of their origin [4].

## MATERIALS AND METHODS

### MSC cultures

Bone marrow was obtained from healthy donors who provided informed consent. We separated cells on a Ficoll density gradient (GE Healthcare, Italy), and the mononuclear cell fraction was collected and washed in phosphate-buffered saline (PBS). We seeded 1–2.5 × 10^5^ cells/cm^2^ in alpha-MEM containing 10% fetal bovine serum (FBS) and basic fibroblast growth factor (bFGF). After 72 hr, non-adherent cells were discarded, and adherent cells were cultivated to confluency. These cells (passage 0) were further amplified to conduct experiments at passages 2–3.

### Culture of muse cells

Bone marrow confluent MSCs were collected by 0.25% trypsin-EDTA and were subjected to cell sorting to isolate Muse cells as described previously [26, 27]. In brief, cells were suspended in FACS Buffer, which contained 0.5% bovine serum albumin (BSA), 2 mM EDTA-2H_2_O in FluoroBrite DMEM (ThermoFisher Scientific, Japan), and were incubated with the anti-human SSEA-3 antibody (1:400, BioLegend, Japan) for 1 hr on ice. Cells were then washed with FACS Buffer for three times and centrifuged at 400 g for 5 min. Subsequently, cells were incubated with a secondary antibody, anti-Rat IgM-FITC (1:100, Jackson ImmunoResearch, PA, USA) for 1 hr on ice, and then washed three times again. Magnetic-activated cell sorting (MACS) was used to collect SSEA-3(+) Muse cells and SSEA-3(−) non-Muse cells according to the manufacturer’s instruction (Miltenyi Biotech, CA, USA). In brief, cells were incubated with anti-FITC microbeads for 15 min in ice, then washed with FACS Buffer. Cells were then loaded on LS columns for magnetic separation.

Collected cells were cultured in 10% FBS, 1 ng/mL bFGF, 2 mM GlutaMAX, kanamycin in low-glucose DMEM for overnight at 37° C 5% CO_2,_ and then they were subjected to analysis.

### Treatment with DNA-damaging agents

Cultures were treated for 1 hr with 300 mM H_2_O_2_. Following treatment, the medium was removed, and a complete medium was added. Cells were then collected for data analysis 1, 6, and 48 hr later. For UV irradiation, cell plates with lids removed were irradiated with UV light by exposure to a germicidal lamp (peak sensitivity approximately 365 nm) in a tissue culture hood (15 mJ/cm^2^) for 7 min. Following treatment, the medium was removed, and a complete medium was added. Cells were collected 1, 6, and 48 hr later.

### Senescence assay

Senescence was evaluated with a quantitative senescence-associated beta-galactosidase assay. Essentially, 4-methylumbelliferyl-β-d-galactopyranoside (4-MUG) is a beta-galactosidase substrate that does not emit fluorescence until cleaved by the enzyme to generate the fluorophore 4-methylumbelliferone. As already reported, we performed an assay on cell lysates to monitor the fluorophore production at an emission/excitation wavelength of 365/460 nm [28].

### Cell death assays

Apoptotic cells were detected using a fluorescein-conjugated Nexin V kit (EMD Millipore, Italy) on a Guava EasyCyte flow cytometer, following the manufacturer’s instruction.

The kit utilizes two separate dyes (Annexin V and 7AAD) to identify a broad spectrum of apoptotic and non-apoptotic cells. Annexin V (red) binds to phosphatidylserine on the external membrane of apoptotic cells, while 7AAD (blue) permeates and stains the DNA of late-stage apoptotic and dead cells. Staining allows the identification of three cell populations: non-apoptotic cells (Annexin V– and 7AAD–); early apoptotic cells (annexin V+ and 7AAD–); and late-apoptotic or dead cells (Annexin V+ and 7AAD+). In our experimental conditions, early and late apoptotic cells were grouped together.

Cell viability was determined with a Quick Cell Proliferation Assay Kit II (Biovision, CA, USA). The assay evaluated the cleavage of a tetrazolium salt (MTT) to formazan by mitochondrial dehydrogenases. The formazan dye, which was produced only by viable cells, was quantified by measuring the absorbance of the dye solution at 440 nm on a microplate reader. After each genotoxic injury, the percentage of surviving and dead cells was determined according to the manufacturer’s instruction.

### Flow cytometry analysis

For cell cycle analysis and immunostaining, cells were collected and fixed in 70% ethanol followed by PBS washes; finally, they were dissolved in a hypotonic buffer containing propidium iodide. Samples were acquired on a Guava EasyCyte flow cytometer (Merck Millipore, Italy) and analyzed with a standard procedure using EasyCyte software. To detect the different proteins involved in DNA repair, we incubated fixed cells with the following antibodies: ATM (ab36810, ABCAM, UK); g-H2AX (2577, Cell Signaling, MA, USA); RAD51 (ab88572, ABCAM, UK); and DNA-PK (sc390698, SantaCruz Biotech, CA, USA). Cells were then incubated with corresponding secondary antibodies that were FITC conjugated. For each sample, 5,000 cells were evaluated on the Guava instrument.

### RNA extraction, RT-PCR, and real-time PCR

We extracted total RNA from cell cultures using Omnizol (EuroClone, Italy) according to the manufacturer’s protocol; we then measured mRNA levels by RT-PCR amplification.

We used sequences of mRNAs from the Nucleotide Data Bank (National Center for Biotechnology Information, MA, USA) to design primer pairs for real-time RT-PCR reactions (Primer Express, Applied Biosystems, Italy); primer sequences are available upon request. We used appropriate regions of HPRT and/or GAPDH cDNA as controls and ran real-time PCR assays on an Opticon 4 machine (MJ Research, MA, USA). We carried out reactions according to the manufacturer’s instruction using an SYBR green PCR master mix and used the 2^-ΔΔCT^ method as a relative quantification strategy for quantitative real-time PCR data analysis.

### Base excision repair (BER) assay

We developed a new method to evaluate the *in vitro* activity of uracil DNA glycosylase that is one of the glycosylases involved in the first step of BER [29]. 1.0–2.0 × 10^6^ cells were lysed in 50 mM Tris-HCl (pH 7.5), 1 M KCl, 2 mM EDTA, and 1 mM DTT. We prepared two batches of cells: naïve and primed. Naïve cells were collected without any previous genotoxic stress; primed cells were UV irradiated. For UV irradiation, cells were plated in cell dishes without lids and irradiated with UV light by exposure to a germicidal lamp (peak sensitivity approximately 365 nm) in a tissue-culture hood (15 mJ/cm^2^). The primed and naïve cell extracts contain active enzymes involved in DNA repair, including the Uracil DNA glycosylase (UDG). Substrates for UDG were obtained by PCR amplification of a human GAPDH DNA segment with a primer pair encompassing a region of the GAPDH gene (gene ID:2597 of NCBI Gene bank; forward primer: 5′-GCATCCTGCACCACCAACTG-3′; reverse primer: 5′-GCCTGCTTCACCACCTTCTT-3′; product size: 347 bp). DNA for PCR amplification was isolated from the human blood sample with a DNeasy Blood & Tissue kit (Qiagen Italia, Italy) according to the manufacturer’s instruction. Isolated DNA was amplified on a Verity Thermal Cycler with AmpliTaq Gold (Thermo Fisher Italia, Italy). We used a dNTP mix containing 2.5 mM of A, C, and G deoxynucleotides, 0.25 mM of T deoxynucleotides, and 5 mM of U deoxynucleotides. Equal amounts of amplified DNA were incubated with 10 µg of each protein extract in a 20 µl of reaction buffer (200 mM HEPES pH 7.5, 800 mM KCl, and 100 mM MgCl_2_) for 3 min at 37° C. Results of reactions were analyzed on 2% agarose gel and then were acquired and quantified with Bio-Rad Chemidoc 2000 (Bio-Rad, CA, USA).

### Nucleotide excision repair (NER) assay

We evaluated the *in vitro* NER activity of primed and naïve cell extracts (obtained as described above). We modified the method of Shen and co-workers [30]. In brief, we amplified with PCR two different DNA templates with a hairpin duplex structure. The templates, named CPD and TT construct, differ only in a region that presents either normal TT dimers (TT construct) or cyclobutane pyrimidine dimers (CPD construct).

In the CPD construct, the primer pair for PCR amplification encompasses a region containing pyrimidine dimers. The presence of these dimers impairs activity of DNA polymerase during PCR unless these lesions are repaired by the NER system. The other DNA template did not contain cyclobutane pyrimidine dimers and served as the PCR amplification control.

Equal amounts CPD or TT construct were incubated with 10 µg of protein extracts obtained from naïve and primed cells. The reaction was performed in a 50 µl of buffer (200 mM HEPES pH 7.5, 800 mM KCl, and 100 mM MgCl_2_) for 60 min at 37° C. Reactions were arrested with the addition of proteinase K and 10% SDS. Then, samples were phenol extracted, ethanol precipitated, and redissolved in 20 µl of DNAse-free water. These samples were amplified on a Verity Thermal Cycler with AmpliTaq Gold (Thermo Fisher Italia, Italy).

### Plasmid-based assay to detect double-strand break DNA repair

This assay was carried out according to the methods described by Diggle *et al.* [31] with modifications. In brief, the p100048 plasmid (Addgene, MA, USA) was digested with an Age I enzyme (New England Biolabs, MT, USA), purified with phenol, and then ethanol precipitated. Cells were lysed in 50 mM Tris-HCl (pH 7.5), 1 M KCl, 2 mM EDTA, and 1 mM DTT. Protein lysates were added to digested and purified p100048 in a reaction buffer (200 mM HEPES pH 7.5, 800 mM KCl, and 100 mM MgCl_2_) that enabled the *in vitro* DNA end-joining of the digested plasmid; the reaction was carried for 2 hr at 37° C. End-joined plasmids were treated with proteinase K and SDS. The reactions were phenol purified, ethanol precipitated, and redissolved in 20 µl of DNAse-free water.

The plasmids were then used as the template for a qPCR reaction that amplifies a 135 bp region. This DNA fragment encompassed the region containing the Age I restriction site and DNA polymerase amplified-only plasmids that had been end-joined. For qPCR, we used the following conditions: forward primer: 5′-ATAACTATACCAGCAGGA-3′; reverse primer: 5′-AATTCAACAGGCATCTAC-3′; iTaq universal SYBR Green Supermix (Bio-Rad, CA, USA). Plasmid samples were amplified on a Verity Thermal Cycler with AmpliTaq Gold (Thermo Fisher Italia, Italy).

### Statistical analysis

We evaluated statistical significance using analysis of variance, followed by the Student’s t and Bonferroni’s tests. For data with continuous outcomes, we used mixed-model variance analysis and, in any case, analyzed all data with the GraphPad Prism version 5.01 (GraphPad, La Jolla, CA, USA).
